# Reductive whole-cell biotransformation with *Corynebacterium glutamicum*: improvement of NADPH generation from glucose by a cyclized pentose phosphate pathway using *pfkA* and *gapA* deletion mutants

**DOI:** 10.1007/s00253-012-4314-7

**Published:** 2012-08-01

**Authors:** Solvej Siedler, Steffen N. Lindner, Stephanie Bringer, Volker F. Wendisch, Michael Bott

**Affiliations:** 1 Institut für Bio–und Geowissenschaften, IBG-1: Biotechnologie, Forschungszentrum Jülich GmbH, 52425 Jülich, Germany; 2 Faculty of Biology & CeBiTec, Bielefeld University, 33615 Bielefeld, Germany

**Keywords:** *Corynebacterium glutamicum*, Pathway engineering, NADPH yield, Pentose phosphate pathway, Resting cells, Reductive whole-cell biotransformation, Phosphofructokinase, Glyceraldehyde 3-phosphate dehydrogenase, *pfk*, *gap*

## Abstract

In this study, the potential of *Corynebacterium glutamicum* for reductive whole-cell biotransformation is shown. The NADPH-dependent reduction of the prochiral methyl acetoacetate (MAA) to the chiral (*R*)-methyl 3-hydroxybutyrate (MHB) by an alcohol dehydrogenase from *Lactobacillus brevis* (*Lbadh*) was used as model reaction and glucose served as substrate for the regeneration of NADPH. Since NADPH is mainly formed in the oxidative branch of the pentose phosphate pathway (PPP), *C. glutamicum* was engineered to redirect carbon flux towards the PPP. Mutants lacking the genes for 6-phosphofructokinase (*pfkA*) or glyceraldehyde 3-phosphate dehydrogenase (*gapA*) were constructed and analyzed with respect to growth, enzyme activities, and biotransformation performance. Both mutants showed strong growth defects in glucose minimal medium. For biotransformation of MAA to MHB using glucose as reductant, strains were transformed with an *Lbadh* expression plasmid. The wild type showed a specific MHB production rate of 3.1 mmol_MHB_ h^−1^ g_cdw_^−1^ and a yield of 2.7 mol_MHB_ mol_glucose_^−1^. The ∆*pfkA* mutant showed a similar MHB production rate, but reached a yield of 4.8 mol_MHB_ mol_glucose_^−1^, approaching the maximal value of 6 mol_NADPH_ mol_glucose_^−1^ expected for a partially cyclized PPP. The specific biotransformation rate of the Δ*gapA* mutant was decreased by 62 % compared to the other strains, but the yield was increased to 7.9 mol_MHB_ mol_glucose_^−1^, which to our knowledge is the highest one reported so far for this mode of NADPH regeneration. As one fourth of the glucose was converted to glycerol, the experimental yield was close to the theoretically maximal yield of 9 mol_NADPH_ mol_glucose_^−1^.

## Introduction

Whole-cell biotransformation has become an important method in chemoenzymatic synthesis, e.g., for the production of amino acids and chiral alcohols (Ishige et al. 2005). *Corynebacterium glutamicum* is a Gram-positive, non-pathogenic soil bacterium which is predominantly used for the large-scale industrial production of the flavor enhancer l-glutamate and the food additive l-lysine (Pfefferle et al. 2003; Kimura 2003; Hermann 2003). Recent metabolic engineering studies have shown that *C. glutamicum* is also capable of producing a variety of other commercially interesting compounds, e.g., other l-amino acids (Wendisch et al. 2006), d-amino acids (Stäbler et al. 2011), organic acids such as succinate (Okino et al. 2008; Litsanov et al. 2012a, b), diamines such as cadaverine (Mimitsuka et al. 2007) or putrescine (Schneider and Wendisch 2010), biofuels such as ethanol or isobutanol (Inui et al. 2004; Smith et al. 2010; Blombach et al. 2011), or proteins (Meissner et al. 2007). An overview of the product spectrum of *C. glutamicum* can be found in a recent review (Becker and Wittmann 2011).

*C. glutamicum* was also shown to be a suitable host for whole-cell biotransformation with resting cells for production of mannitol (Bäumchen and Bringer-Meyer 2007) and cyclohexanone derivatives (Doo et al. 2009; Yun et al. 2012). These reactions are often NAD(P)H dependent and cofactor recycling is crucial for profitable processes. For example, formate dehydrogenase or glucose dehydrogenase are used, but only 1 mol NAD(P)H can be generated from 1 mol formate or 1 mol glucose (Kaup et al. 2004, 2005; Ernst et al. 2005; Eguchi et al. 1992; Tan 2006). Use of metabolically active cells gives the opportunity to regenerate reduced cofactors via sugar metabolism and to gain a higher reduced cofactor to glucose ratio (Chin and Cirino 2011).

**Fig. 1:**
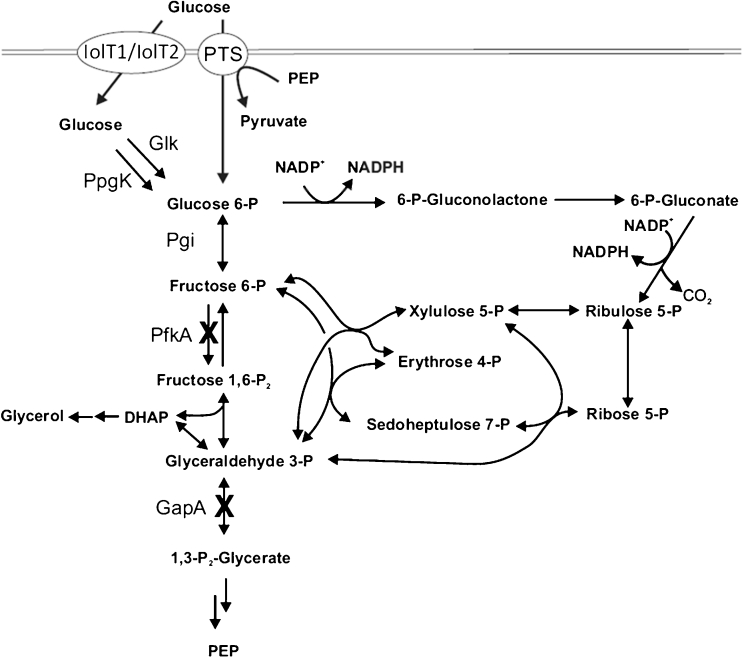
Scheme of the upper part of glycolysis and pentose phosphate pathway of *C. glutamicum*. Gene deletions and NADPH generating reactions are indicated. *PTS* phosphotransferase system, *IolT1/IolT2* alternative glucose import system, *Glk* ATP-dependent glucokinase, *PpgK* polyphosphate/ATP-dependent glucokinase, *Pgi* phosphoglucose isomerase, *PfkA* phosphofructokinase, *GapA* glyceraldehyde-3-phosphate dehydrogenase, *DHAP* dihydroxyacetone phosphate, *PEP* phosphoenolpyruvate

In *Escherichia coli*, several attempts were made for engineering cellular metabolism towards a higher NADPH per glucose yield (Fasan et al. 2011; Akinterinwa and Cirino 2011). NADPH is mainly generated in the oxidative branch of the pentose phosphate pathway (PPP), where glucose 6-phosphate dehydrogenase catalyzes the oxidation of glucose 6-phosphate to 6-phopshoglucono-δ-lactone and 6-phosphogluconate dehydrogenase, which catalyzes the oxidative decarboxylation of 6-phosphogluconate to ribulose 5-phosphate, yielding 2 mol NADPH (Fig. 1). Therefore, employment of the PPP is an interesting option for NADPH-dependent processes (Chin and Cirino 2011; Chemler et al. 2010). In a recent study with *E. coli*, we analyzed the NADPH-dependent reduction of the prochiral β-ketoester methyl acetoacetate (MAA) to the chiral hydroxy ester (*R*)-methyl 3-hydroxybutyrate (MHB) using glucose as substrate for the generation of NADPH (Siedler et al. 2011, 2012). The reduction was catalyzed by an *R*-specific alcohol dehydrogenase (ADH) from *Lactobacillus brevis*. MHB serves as a building block of statins (Panke and Wubbolts 2005). Deletion of *pfkA* and *pfkB* encoding phosphofructokinase I and II, respectively, resulted in a partial cyclization of the PPP and a yield of 5.4 mol_MHB_ mol_glucose_^−1^, which was near the theoretically maximal yield of 6 (Kruger and von Schaewen 2003).

To determine whether this metabolic engineering strategy can be generalized, is e.g. transferable to *C. glutamicum*, was one major goal of this study. It has to be kept in mind that differences exist in the repertoires of metabolic enzymes of *E. coli* and *C. glutamicum*. Of relevance for the present work is the occurrence of only one gene encoding a 6-phosphofructo1-kinase (*pfkA*) and the absence of genes encoding transhydrogenases and the key enzymes of the Entner–Doudoroff-pathway in *C. glutamicum* (Yokota and Lindley 2005)*.* To further improve the NADPH per glucose yield, deletion of the glyceraldehyde 3-phosphate dehydrogenase (*gapA*) gene would be beneficial, as it should result in a complete cyclization of the PPP. Deletion of *gapA* theoretically enables a yield of 12 mol NADPH per mole of glucose 6-phosphate by complete recycling of fructose 6-phosphate and triose 3-phosphate through the oxidative PPP (Kruger and von Schaewen 2003). The *gapB* gene encoding a second glyceraldehyde 3-phosphate dehydrogenase in *C. glutamicum* should not be relevant in this context, as GapB does not function in the glycolytic direction (Omumasaba et al. 2004).

In this study, we analyzed *C. glutamicum* mutants lacking either *pfkA* or *gapA* for their behavior in reductive whole-cell biotransformation. The results supported the view that the PPP operates in cyclic manner, oxidizing glucose to CO_2_ with concomitant reduction of NADP^+^ to NADPH.

## Materials and methods

### Chemicals and enzymes

Chemicals were obtained from Sigma-Aldrich (Taufkirchen, Germany), Qiagen (Hilden, Germany), Merck (Darmstadt, Germany), and Roche Diagnostics (Mannheim, Germany).

### Bacterial strains, plasmids, media, and growth conditions

Strains and plasmids used in this work are listed in Table 1. *E. coli* strains were transformed by the method described by Hanahan (1983) and cultivated in LB medium (Miller 1972). *E. coli* DH5α was used for cloning purposes and *C. glutamicum* ATCC 13032 and derivatives for gene expression and whole-cell biotransformation. When required, antibiotics were added to the medium at a final concentration of 50 μg kanamycin ml^−1^ (pEKEx2-LbADH) or 100 μg spectinomycin ml^−1^ (pEKEx3 derivatives).

**Table 1:** Strains and plasmids used in this work

Strains and plasmids	Relevant characteristics	Reference
Strains		
*E. coli* DH5α	F^−^ ø80∆*lacZ*∆M15 ∆(*lacZYA-argF*) U169 *deoR recA*1 *endA*1 *hsdR*17 (rk^−^, mk^+^) *phoA supE*44 λ^−^ *thi*-1 *gyrA*96 *relA*1	(Hanahan 1983), Invitrogen
*C. glutamicum* ATCC13032	Wild type, biotin auxotrophic	(Abe et al. 1967)
∆*pfkA*	*C. glutamicum* ATCC13032 ∆*pfkA* (cg1409)	This study
∆*gapA*	*C. glutamicum* ATCC13032 ∆*gapA* (cg1791)	This study
WT/pEKEx3	*C. glutamicum* ATCC13032 with pEKEx3	This study
WT/pEKEx3-*pfkA* ^Cgl^	*C. glutamicum* ATCC13032 with pEKEx3-*pfkA* ^Cgl^	This study
WT/pEKEx3-*pfkA* ^Eco^	*C. glutamicum* ATCC13032 with pEKEx3-*pfkA* ^Eco^	This study
WT/pEKEx3-*pfkB* ^Eco^	*C. glutamicum* ATCC13032 with pEKEx3-*pfkB* ^Eco^	This study
WT/pEKEx3-*gapA* ^Cgl^	*C. glutamicum* ATCC13032 with pEKEx3-*gapA* ^Cgl^	This study
WT/pEKEx2-*Lbadh*	*C. glutamicum* ATCC13032 with pEKEx2-*Lbadh*	This study
∆*pfkA*/pEKEx3	*C. glutamicum* ATCC13032 ∆*pfkA* with pEKEx3	This study
∆*pfkA*/pEKEx3-*pfkA* ^Cgl^	*C. glutamicum* ATCC13032 ∆*pfkA* with pEKEx3-*pfkA* ^Cgl^	This study
∆*pfkA*/pEKEx3-*pfkA* ^Eco^	*C. glutamicum* ATCC13032 ∆*pfkA* with pEKEx3-*pfkA* ^Eco^	This study
∆*pfkA*/pEKEx3-*pfkB* ^Eco^	*C. glutamicum* ATCC13032 ∆*pfkA* with pEKEx3-*pfkB* ^Eco^	This study
∆*pfkA*/pEKEx2-*Lbadh*	*C. glutamicum* ATCC13032 ∆*pfkA* with pEKEx2-*Lbadh*	This study
∆*gapA*/pEKEx3	*C. glutamicum* ATCC13032 ∆*gapA* with pEKEx3	This study
∆*gapA*/pEKEx3-*gapA* ^Cgl^	*C. glutamicum* ATCC13032 ∆*gapA* with pEKEx3-*gapA* ^Cgl^	This study
∆*gapA*/pEKEx2-*Lbadh*	*C. glutamicum* ATCC13032 ∆*gapA* with pEKEx2-*Lbadh*	This study
Plasmids		
pEKEx2	Kan^r^; *E. coli*–*C. glutamicum* shuttle vector for regulated gene expression (P_tac_ *lacI* ^q^ pBL1 *oriV*_*C.g.*_ pUC18 *oriV*_*E.c.*_ *)*	(Eikmanns et al. 1991)
pEKEx2-*Lbadh*	Kan^r^; pEKEx2 derivative with *adh* gene from *Lactobacillus brevis*	This study
pEKEx3	Spec^r^; *C. glutamicum*/*E. coli* shuttle vector (P_tac_, *lacI* ^q^; pBL1, *oriV*_*C.g.*_, *oriV*_*E.c.*_)	(Stansen et al. 2005)
pEKEx3-*pfkA* ^Cgl^	Spec^r^; derivative of pEKEx3 for regulated expression of *pfkA* (cg1409) of *C. glutamicum*	This study
pEKEx3-*gapA* ^Cgl^	Spec^r^; derivative of pEKEx3 for regulated expression of *gapA* (cg1791) of *C. glutamicum*	This study
pEKEx3-*pfkA* ^Eco^	Spec^r^; derivative of pEKEx3 for regulated expression of *pfkA* (b3916) of *E. coli*	This study
pEKEx3-*pfkB* ^Eco^	Spec^r^; derivative of pEKEx3 for regulated expression of *pfkB* (b1723) of *E. coli*	This study
pK19*mobsacB*	Kan^r^; mobilizable *E. coli* vector used for the construction of *C. glutamicum* insertion and deletion mutants (RP4 *mob*; *sacB*_*B.sub.*_; *lacZα*; *oriV*_*E.c.*_)	(Schäfer et al. 1994)
pK19*mobsacB*∆*pfkA*	Kan^r^; pK19*mobsacB* derivative containing a PCR product which covers the flanking regions of the *C. glutamicum pfkA* (cg1409) gene	This study
pK19*mobsacB*∆*gapA*	Kan^r^; pK19*mobsacB* derivative containing a PCR product which covers the flanking regions of the *C. glutamicum gapA* (cg1791) gene	This study

For growth experiments with *C. glutamicum*, 50-ml LB overnight cultures were inoculated from LB plates, harvested by centrifugation (10 min, 3,220 × *g*), washed in CgXII medium (Eggeling and Bott 2005), and inoculated in CgXII medium containing 100 mM glucose to a final optical density at 600 nm (OD_600_) of 1. When appropriate, 1 mM isopropyl-β-d-thiogalactopyranosid (IPTG), 25 μg ml^−1^ kanamycin, and 100 μg ml^−1^ spectinomycin was added. For all growth experiments, 500 ml baffled shake flasks with 50 ml CgXII medium were used and incubated at 30 °C and 120 rpm. Growth was followed by OD_600_ determination using a UV-1650 PC photometer (Shimadzu, Duisburg, Germany). The biomass concentration was calculated from OD_600_ values using an experimentally determined correlation factor of 0.25 g (dry weight) of cells (cdw) per liter for an OD_600_ of 1 (Kabus et al. 2007). For the determination of enzyme activity in cell-free extracts, 50 ml LB medium containing 1 mM IPTG and 100 μg ml^−1^ spectinomycin was inoculated from LB overnight cultures to an OD_600_ of 0.5. At an OD_600_ of 4, cells were harvested by centrifugation (10 min, 3,220 × *g*, 4 °C) and stored at −20 °C until use.

### Recombinant DNA work

Standard methods like polymerase chain reaction (PCR), restriction, or ligation were carried out according to established protocols (Sambrook and Russell 2001). *E. coli* cells were transformed by the CaCl_2_ method (Hanahan et al. 1991). DNA sequencing was performed by Eurofins MWG Operon (Germany). Oligonucleotides (listed in Table 2) were synthesized by Biolegio bv (Nijmegen, The Netherlands) and Eurofins MWG Operon (Germany).

**Table 2:** Sequences of oligonucleotide primers

Name	Sequence (5′–3′)	Function and relevant characteristics
pfkA-cgl-fw	**GGATCC** *GAAAGGAGG*CCCTTCAGATGGAAGACATGCGAATTGCTAC	OE of Cgl *pfkA*; start; ***BamH*** **i**; *RBS*
pfkA-cgl-rv	**GGATCC** CTATCCAAACATTGCCTGGGC	OE of Cgl *pfkA*; stop; ***BamH*** **i**
gapA-cgl-fw	*AAGGAGA*TATAGATATGACCATTCGTGTTGGTATTAAC	OE of Cgl *gapA*; start; *RBS*
gapA-cgl-rv	TTAGAGCTTGGAAGCTACGAGCTC	OE of Cgl *gapA*; stop
pfkA-eco-fw	CC**GGATCC** *GAAAGGAGG*CCCTTCAGATGATTAAGAAAATCGGTGTGTTGAC	OE of Eco *pfkA*; start; ***BamH*** **I**; *RBS*
pfkA-eco-rv	CC**GGATCC** TTAATACAGTTTTTTCGCGCAGTC	OE of Eco *pfkA*; stop; ***BamH*** **I**
pfkB-eco-fw	GA**CTGCAG** *GAAAGGAGG*CCCTTCAGATGGTACGTATCTATACGTTGACAC	OE of Eco *pfkB*; start; ***Pst*** **I**; *RBS*
pfkB-eco-rv	GG**CTGCAG** TTAGCGGGAAAGGTAAGCGTAA	OE of Eco *pfkB*; stop; ***Pst*** **I**
pfkA-Del-A	CCGGAATATCTCGACGCCACAGAACGC	Del of *pfkA*
pfkA-Del-B	*CCCATCCACTAAACTTAAACA*AATTCGCATGTCTTCCATATTAAACCCATCACAACACCCGC	Del of *pfkA*; *linker sequence*
pfkA-Del-C	*TGTTTAAGTTTAGTGGATGGG*GAACGCTGGGTTACTGCCCAGGCAATGTTT	Del of *pfkA*; *linker sequence*
pfkA-Del-D	CCGAAGGAATAGACGAGTTAACAAAACTACGGTCTG	Del of *pfkA*
pfkA-Del-Ver-fw	GCCAAAACTCGAGTAGCCCGG	Verification of *pfkA* Del
pfkA-Del-Ver-rv	CCACAGCTTCAGTCATGCCC	Verification of *pfkA* Del
gapA-Del-A	GGCTGATCCTCAAATGACCAAG	Del of *gapA*
gapA-Del-B	*CCCATCCACTAAACTTAAACA*ACCAACACGAATGGTCATGTTG	Del of *gapA*; *linker sequence*
gapA-Del-C	*TGTTTAAGTTTAGTGGATGGG*CTGCGTCTGACCGAGCTCGTAG	Del of *gapA*; *linker sequence*
gapA-Del-D	CACCGAAGCCGTCAGAAACGAATG	Del of *gapA*
gapA-Del-Ver-fw	CCAACTTCGACGATGCCAATC	Verification of *gapA* Del
gapA-Del-Ver-rv	CTCTGGTGATTCTGCGATCTTTTC	Verification of *gapA* Del
lbADH_for	CAGT**GGATCC** *GAAAGGAGG*CCCTTCAGATGTCTAACCGTTTGGATGG	OE of *Lb adh*; start; ***BamH*** **I**; *RBS*
lbADH_rev	GTCT**GAATT** **C** TATTGAGCAGTGTAGCCACC	OE of *Lb adh*; stop; ***EcoRI***

Restriction sites are highlighted in bold; linker sequences for crossover PCR and ribosomal binding sites are shown in italics; stop and start codons are underlined

*OE* overexpression, *Del* deletion, *RBS* ribosomal binding site, *Cgl C. glutamicum*, *Eco E. coli*

### Construction of deletion mutants and plasmids

*C. glutamicum* deletion mutants were constructed using pK19*mobsacB* (Schäfer et al. 1994) using the procedure described by Niebisch and Bott (2001). Upstream and downstream flanking regions of *pfkA* (cg1409), and *gapA* (cg1791) were amplified by PCR using the oligonucleotide pairs pfkA-Del-A/pfkA-Del-B and pfkA-Del-C/pfkA-Del-D for deletion of *pfkA*, and gapA-Del-A/gapA-Del-B and gapA-Del-C/gapA-Del-D for deletion of *gapA* (see Table 2 for primer sequences). The upstream and downstream flanking regions of each gene were fused by overlap extension PCR, resulting in a DNA fragment of about 1 kb. The resulting PCR products were cloned into SmaI-restricted vector pK19*mobsacB* resulting in pK19*mobsacB*∆*pfkA*, and pK19*mobsacB*∆*gapA*. The correctness of the cloned PCR fragments was confirmed by DNA sequencing. Transformation of *C. glutamicum* wild type with these plasmids and selection for the first and second homologous recombination was performed as described (Niebisch and Bott 2001; Rittmann et al. 2003). Kanamycin-sensitive and sucrose-resistant clones were analyzed by PCR using oligonucleotide pairs pfkA-Del-Ver-fw/pfkA-Del-Ver-rv or gapA-Del-Ver-fw/gapA-Del-Ver-rv.

For the complementation of deletion mutants, the genes *pfkA* (cg1409), and *gapA* (cg1791) from *C. glutamicum* and the genes *pfkA* (b3916) and *pfkB* (b3916) from *E. coli* were amplified via PCR from genomic DNA of *C. glutamicum* WT, which was prepared as described previously (Eikmanns et al. 1995), and *E. coli* MG1655 genomic DNA, which was prepared by using the DNA isolation kit (Roche, Mannheim, Germany). PCR was performed using the following oligonucleotide pairs: pfkA-cgl-fw/pfkA-cgl-rv, gapA-cgl-fw/gapA-cgl-rv, pfkA-eco-fw/pfkA-eco-rv, and pfkB-eco-fw/pfkB-eco-rv (see Table 2). To allow IPTG-inducible expression of *pfkA*, and *gapA* from *C. glutamicum* and *pfkA*, and *pfkB* from *E. coli* the corresponding PCR products were ligated into the SmaI*-*restricted vector pEKEx3 resulting in pEKEx3-*pfkA* ^Cgl^, pEKEx3-*gapA* ^Cgl^, pEKEx3-*pfkA* ^Eco^, and pEKEx3-*pfkB* ^Eco^.

For the construction of the expression plasmid pEKEx2-*Lbadh*, the *adh* gene of *L. brevis* was amplified together with a 9-bp linker and an artificial ribosome binding site (AAGGAG) using the oligonucleotides Lbadh_for and Lbadh_rev and the plasmid pBtac*Lbadh* as template (Ernst et al. 2005). The PCR product was digested with *BamH*I and *EcoR*I and cloned into the vector pEKEx2. The correctness of the cloned PCR fragments in the plasmids was confirmed by DNA sequencing.

### Enzyme activity assays

For the determination of alcohol dehydrogenase activity, cells were harvested by centrifugation (10,000 × *g*, 4 °C, 5 min) 30 min after start of biotransformation and stored at −20 °C until use. The cells were resuspended in 100 mM potassium phosphate buffer, pH 6.5, with 1 mM dithiothreitol and 1 mM MgCl_2_. Cells were disrupted at 4 °C by 3 × 15 s bead-beating with 0.1-mm-diameter glass beads using a Silamat S5 (Ivoclar Vivadent GmbH, Germany) and crude extracts were centrifuged at 16,000 × *g* (4 °C, 20 min) to remove intact cells and cell debris. The supernatants were used as cell-free extracts. Alcohol dehydrogenase activity was determined photometrically at 340 nm using a mixture of 10 mM methyl acetoacetate, 250 μM NADPH, and 1 mM MgCl_2_ in 100 mM potassium phosphate buffer, pH 6.5. The reactions were started by adding different dilutions of the cell-free extract. For rate calculation, an extinction coefficient for NADPH at 340 nm of 6.22 mM^−1^ cm^−1^ was used. One unit of enzyme activity corresponds to 1 μmol NADPH consumed per minute.

For the determination of the specific activity of phosphofructokinase and glyceraldehyde 3-phosphate dehydrogenase, cells were harvested by centrifugation (3,220 × *g*, 4 °C, 10 min) and washed in the appropriate buffer (see below) and stored at −20 °C until use. Cells were resuspended in 1 ml of the buffer and cell-free extracts were prepared by sonification as described previously (Stansen et al. 2005). All enzyme activity measurements were carried out at 30 °C. Protein concentrations were determined with bovine serum albumin as standard using Bradford reagents (Sigma, Taufkirchen, Germany).

6-Phosphofructokinase activity was measured spectrophotometrically at 340 nm according to Babul (1978) by a coupled enzymatic assay with pyruvate kinase and lactate dehydrogenase. ADP formed in kinase reaction was used to convert phosphoenolpyruvate to pyruvate, which was subsequently reduced to lactate with concomitant oxidation of NADH to NAD^+^. The assay solution contained 100 mM Tris–HCl pH 7.5, 0.2 mM NADH, 1 mM ATP, 10 mM MgCl_2_, and 0.2 mM phosphoenolpyruvate. One unit of enzyme activity corresponds to 1 μmol NADH oxidized per minute.

Glyceraldehyde 3-phosphate dehydrogenase activity was measured according to Omumasaba et al. (2004). The assay contained 1 mM NAD^+^, 50 mM Na_2_HPO_4_, 0.2 mM EDTA, and 0.5 mM glyceraldehyde 3-phosphate in 50 mM triethanolamine hydrochloride (TEA) buffer pH 8.5. One unit of enzyme activity corresponds to 1 μmol NADH formed per minute.

### Whole-cell biotransformation

For cultivation of the different recombinant *C. glutamicum* strains carrying the pEKEx2-*Lbadh* plasmid, a single colony of each strain was inoculated into 10 ml BHIS medium (37 g l^−1^ brain heart infusion, 91 g l^−1^ sorbitol) containing the appropriate selection marker as described above and grown overnight at 30 °C and 120 rpm. These pre-cultures were used for inoculation of the main cultures to an optical density at 600 nm (OD_600_) of 0.4. Main cultures were grown in 100 ml BHIS medium in shake flasks in the presence of the appropriate selection marker and 0.5 mM IPTG at 30 °C and 120 rpm. The cells were harvested at an OD_600_ between 2.5 and 5 by centrifugation (4,000 × *g*, 4 °C, 7 min) and resuspended in a solution containing 111 mM glucose, 2 mM MgSO_4_, and 250 mM potassium phosphate buffer, pH 6.5, to a cell density of 3 g_cdw_ l^−1^. The biotransformation was started by adding 50 mM MAA and conducted in shake flasks at 30 °C and 120 rpm to prevent cell sedimentation. Specific productivities (mmol_MHB_ h^−1^ g_cdw_^−1^) were determined by taking samples at 30–60-min time intervals over a period of 3 h. MHB and glucose concentrations of the samples were determined (see below). Specific productivities were calculated by dividing the slope of graphs showing MHB concentration vs. time by the cell dry weight, which remained constant.

### Analysis of substrates and products

Methyl acetoacetate (MAA), (*R*)-methyl 3-hydroxybutyrate (MHB), glucose, and extracellular metabolites were analyzed by HPLC as described previously (Siedler et al. 2011).

## Results

### Growth behavior and in vitro enzyme activities of *C. glutamicum* wild-type and mutant strains

In a *C. glutamicum* mutant lacking 6-phosphofructokinase, glucose catabolism is forced to proceed via the pentose phosphate pathway. Fructose 6-phosphate formed in the PPP by transaldolase or transketolase has to re-enter the oxidative part of the PPP again and only glyceraldehyde 3-phosphate can be catabolized further via the lower part of the glycolytic pathway. Thus, the initial part of glucose catabolism in a ∆*pfkA* mutant can be described by the following equation: Glucose 6-phosphate + 6 NADP^+^ ➔ Glyceraldehyde 3-phosphate + 3 CO_2_ + 6 NADPH + 6 H^+^. Thus, 6 mol NADPH are formed per mole of glucose.

The deletion of the *pfkA* gene prevented growth in CgXII medium with 100 mM glucose (Table 3). The growth defect of the ∆*pfkA* mutant was complemented to levels of the WT control (0.32 h^−1^) by plasmid-based overexpression of either the homologous *pfkA* gene from *C. glutamicum* (0.32 h^−1^) or of the heterologous *pfkA* gene from *E. coli* (0.33 h^−1^) and increased to 0.16 h^−1^ by heterologous expression of *pfkB* from *E. coli*. The slow growth of ∆*pfkA*/pEKEx3-*pfkB* ^Eco^ was accompanied by a significantly higher biomass yield of 10.8 g l^−1^ compared to 8.4 g l^−1^ of WT/pEKEx3 or 8.6 g l^−1^ of strain Δ*pfkA*/pEKEx3-*pfkA* ^Cgl^.

**Table 3:** Growth rates (*μ*) and biomass concentrations [cell dry weight (cdw) l^−1^] in glucose minimal medium with 1 mM IPTG and 100 μg ml^−1^ spectinomycin, and specific phosphofructokinase (Pfk) activity in cell extracts of the indicated *C. glutamicum* strains after cultivation in LB medium with 1 mM IPTG and 100 μg ml^−1^ spectinomycin

*C. glutamicum*	*μ* (h^−1^)	cdw (g l^−1^)^a^	Pfk activity (μmol min^−1^ mg^−1^)
WT/pEKEx3	0.32 ± 0.00	8.43 ± 0.18	0.04 ± 0.01
WT/pEKEx3-*pfkA* ^Cgl^	0.30 ± 0.00	8.13 ± 0.07	0.12 ± 0.02
WT/pEKEx3-*pfkA* ^Eco^	0.32 ± 0.00	7.53 ± 0.02	0.11 ± 0.02
WT/pEKEx3-*pfkB* ^Eco^	0.32 ± 0.00	8.48 ± 0.03	0.19 ± 0.02
∆*pfkA*/pEKEx3	0.00 ± 0.00	0.14 ± 0.01^b^	0.00 ± 0.00
∆*pfkA*/pEKEx3-*pfkA* ^Cgl^	0.32 ± 0.01	8.63 ± 0.07	0.10 ± 0.01
∆*pfkA*/pEKEx3-*pfkA* ^Eco^	0.33 ± 0.00	7.93 ± 0.33	0.10 ± 0.02
∆*pfkA*/pEKEx3-*pfkB* ^Eco^	0.16 ± 0.00	10.80 ± 0.10	0.13 ± 0.01

^a^Determination of cdw at maximal biomass

^b^Determination of cdw after 24 h

6-Phosphofructokinase activity was absent in the *pfkA* deletion strain (Table 3). Plasmid-borne expression of *C. glutamicum pfkA* or of *E. coli pfkA* or *pfkB* increased phosphofructokinase activity in the WT background from 0.04 U mg^−1^ to 0.12, 0.11, and 0.19 U mg^−1^, respectively. In the ∆*pfkA* background, phosphofructokinase activities of 0.10 to 0.13 U mg^−1^ were determined when either *C. glutamicum pfkA* or *E. coli pfkA* or *pfkB* was overexpressed (Table 3).

*C. glutamicum* possesses two glyceraldehyde 3-phosphate dehydrogenases, GapA and GapB, but only GapA functions in the glycolytic direction as a Δ*gapA* deletion mutant was unable to grow in glucose minimal medium whereas a Δ*gapB* mutant showed no growth defect under these conditions (Omumasaba et al. 2004). A complete block of glyceraldehyde 3-phosphate conversion to 1,3-bisphosphoglycerate should lead to a complete oxidation of glucose in the PPP according to the equation: Glucose + 6 H_2_O + 12 NADP^+^ ➔ 6 CO_2_ + 12 NADPH + 12 H^+^.

In agreement with previous results (Omumasaba et al. 2004), a deletion of the *gapA* gene in strain ATCC 13032 resulted in an inability to grow in glucose minimal medium. This defect was complemented by plasmid-based overexpression of the *gapA* gene. NAD^+^-dependent glyceraldehyde-3-phosphate dehydrogenase activity of cell-free extracts was 0.15 U mg^−1^ in WT/pEKEx3 and absent in strain ∆*gapA*/pEKEx3. In strains WT/pEKEx3-*gapA* and ∆*gapA*/pEKEx3-*gapA*, the glyceraldehyde 3-phosphate dehydrogenase activity with NAD^+^ was found to be 0.26 and 0.13 U mg^−1^, respectively (Table 4).

**Table 4:** Growth rates (*μ*) and biomass concentrations [cell dry weight (cdw) l^−1^] in glucose minimal medium with 1 mM IPTG and 100 μg ml^−1^ spectinomycin, and specific NAD^+^-dependent glyceraldehyde 3-phosphate dehydrogenase (GAPDH) activity in cell extracts of the indicated *C. glutamicum* strains after cultivation in LB medium with 1 mM IPTG and 100 μg ml^−1^ spectinomycin

*C. glutamicum*	*μ* (h^−1^)	cdw (g l^−1^)^a^	GAPDH activity (μmol min^−1^ mg^−1^)
WT/pEKEx3	0.33 ± 0.01	7.80 ± 0.07	0.15 ± 0.02
WT/pEKEx3-*gapA* ^Cgl^	0.31 ± 0.00	8.08 ± 0.11	0.26 ± 0.03
∆*gapA*/pEKEx3	0.00 ± 0.01	0.00 ± 0.00^b^	0.00 ± 0.00
∆*gapA*/pEKEx3-*gapA* ^Cgl^	0.27 ± 0.01	7.99 ± 0.30	0.13 ± 0.02

^a^Determination of cdw at maximal biomass

^b^Determination of cdw after 24 h

### Biotransformation of MAA to MHB with the reference and the mutant strains

For biotransformation of MAA to MHB, the gene encoding the (*R*)-specific alcohol dehydrogenase of *L. brevis* (*Lbadh*) was overexpressed in *C. glutamicum* WT and in the deletion strains ∆*pfkA* and ∆*gapA* using plasmid pEKEx2-*Lbadh*. The specific NADPH-dependent MAA dehydrogenase activity in cell-free extracts of these strains was similar, ranging from 0.51 to 0.76 U mg^−1^ in independent experiments. Assuming that the in vivo activities are comparable, they are not limiting the biotransformation rate. The *C. glutamicum* wild type showed a MAA dehydrogenase activity below 0.01 U mg^−1^ with either NADPH or NADH as cofactor indicating that the biotransformation occurred only in the presence of the recombinant ADH from *L. brevis.* For the biotransformation, the strains were cultivated in BHIS medium to the exponential growth phase and then harvested and resuspended in 250 mM potassium phosphate buffer pH 6.5 containing 111 mM glucose and 2 mM MgSO_4_ to a cell density of 3 g_cdw_ l^−1^. The resulting cell suspensions were incubated at 30 °C and 120 rpm and the biotransformation was started by adding 50 mM MAA.

**Fig. 2:**
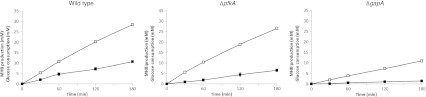
Kinetics of MHB production (*open squares*) and glucose consumption (*filled squares*) during biotransformation of MAA to MHB using resting cells (3 g_cdw_ l^−1^) of the indicated *C. glutamicum* strains carrying the plasmid pEKEx2-*Lbadh*. The cell suspensions were incubated at 30 °C and 120 rpm. Mean values and standard deviations from three independent experiments are shown

The kinetics of MHB production and of glucose consumption of the wild-type and the two mutant strains carrying pEKEx2-*Lbadh* over a period of 180 min are shown in Fig. 2, and the rates and yields are listed in Table 5. It is evident from Fig. 2 that the rates of MHB production and glucose consumption were almost constant within the time period investigated and proportional to each other. The strain WT/pEKEx2-*Lbadh* showed an MHB production rate of 3.14 mmol h^−1^ g_cdw_^−1^ and a glucose consumption rate of 1.17 mmol h^−1^ g_cdw_^−1^. This resulted in a MHB yield of 2.7 mol per mole of glucose, corresponding to an NADPH yield of 2.7 mol per mole of glucose. The strain Δ*pfkA*/pEKEx2-*Lbadh* had an 8 % reduced MHB production rate and a 49 % reduced glucose consumption rate, resulting in a 78 % increased MHB yield of 4.8 mol per mole of glucose. The strain Δ*gapA*/pEKEx2-*Lbadh* showed a 62 % decreased MHB production rate and an 87 % reduced glucose consumption rate, corresponding to a 193 % increase of the MHB yield of 7.9 mol per mole of glucose. As discussed below, the strongly reduced glucose uptake rate of the strain Δ*gapA*/pEKEx2-*Lbadh* is most likely a consequence of the fact that the strain does not form PEP.

**Table 5:** Biotransformation parameters and by-product formation of *C. glutamicum* wild-type and deletion mutants carrying plasmid pEKEx2-*Lbadh*

*C. glutamicum* strain	Specific MHB production rate	Specific glucose consumption rate	Yield	Specific acetate formation rate	Specific succinate formation rate	Specific glycerol formation rate
	(mmol h^−1^ g_cdw_^−1^)	(mmol h^−1^ g_cdw_^−1^)	(mol_MHB_ mol_Glucose_^−1^)	(mmol h^−1^ g_cdw_^−1^)	(mmol h^−1^ g_cdw_^−1^)	(mmol h^−1^ g_cdw_^−1^)
WT/pEKEx2-*Lbadh*	3.14 ± 0.13	1.17 ± 0.07	2.7 ± 0.1	1.19 ± 0.01	0.19 ± 0.01	0
∆*pfkA*/pEKEx2-*Lbadh*	2.88 ± 0.08	0.60 ± 0.01	4.8 ± 0.2	0.05 ± 0.01	0	0
∆*gapA*/pEKEx2-*Lbadh*	1.20 ± 0.04	0.15 ± 0.03	7.9 ± 0.9	0	0	0.08 ± 0.04

### By-product formation of wild-type and mutant strains

During biotransformation, by-product formation was nearly constant and specific rates were calculated (Table 5). The strain WT/pEKEx2-*Lbadh* showed an acetate formation rate (1.19 mmol h^−1^ g_cdw_^−1^) comparable to the glucose consumption rate (1.17 mmol h^−1^ g_cdw_^−1^). In addition, WT/pEKEx2-*Lbadh* formed succinate as by-product with a rate of 0.19 mmol h^−1^ g_cdw_^−1^. A low acetate production rate of 0.05 mmol h^−1^ g_cdw_^−1^ was shown by the strain Δ*pfkA*/pEKEx2-*Lbadh*, which corresponds to only 8 % of the glucose uptake rate. Succinate was not formed by Δ*pfkA*/pEKEx2-*Lbadh*. The strain Δ*gapA*/pEKEx2-*Lbadh* formed neither acetate nor succinate, but glycerol with a rate of 0.08 mmol h^−1^ g_cdw_^−1^, which corresponds to 53 % of the glucose consumption rate. As glyceraldehyde 3-phosphate cannot be catabolized to pyruvate in the Δ*gapA* mutant, reduction to glycerol presents an alternative pathway to oxidation in the cyclic PPP.

## Discussion

For reductive whole-cell biotransformations requiring NADPH, attempts were made in this work to increase the NADPH yield per mole of glucose using *C. glutamicum* as host strain and the reduction of MAA to MHB as NADPH-requiring model reaction. Rerouting of glucose catabolism from glycolysis to the oxidative PPP was achieved by deletion of either the *pfkA* gene or the *gapA* gene.

*C. glutamicum* wild type carrying pEKEx2-*Lbadh* showed a 31 % lower specific MHB production rate compared to *E. coli* carrying pBtac-*Lbadh*, even when compared to an *E. coli* biotransformation conducted at 30 °C (unpublished data). This difference might be due to a lower glucose uptake capacity or to a generally lower metabolic flux capacity of *C. glutamicum*. Overexpression of the genes involved in glucose uptake and catabolism via glycolysis or PPP could improve the rate of glucose catabolism, as shown recently for oxygen-deprived conditions (Yamamoto et al. 2012; Jojima et al. 2010). The MHB per glucose yield found for *C. glutamicum* WT/pEKEx2-*Lbadh* (2.7 mol/mol) was 10 % higher than the corresponding value determined for *E. coli* BL21(DE3)/pBtac-*Lbadh* (2.44 mol/mol) (Siedler et al. 2011), which might be due to slight differences in the partition of glucose 6-phosphate between glycolysis and the PPP.

Biotransformation studies with *E. coli* ∆*pfkA* and ∆*pfkA*∆*pfkB* mutants expressing *Lbadh* showed yields of 4.8 and 5.4 mol_MHB_ mol_glucose_^−1^, respectively (Siedler et al. 2011). ^13^C metabolic flux analysis demonstrated a negative net flux through phosphoglucose isomerase in the ∆*pfkA* mutant, in compliance with the proposed partial cyclization of the PPP (Siedler et al. 2012). The MHB yield per glucose of the *E. coli* strain ∆*pfkA*/pBtac-*Lbadh* was comparable to that of the *C. glutamicum* strain ∆*pfkA*/pEKEx2-*Lbadh* (4.8 mol_MHB_ mol_glucose_^−1^), indicating that a partial cyclization of the PPP occurred in the latter species, too. Furthermore, similarities were found when comparing by-product formation in *E. coli* and *C. glutamicum*. Less acetate and no succinate was produced in both ∆*pfkA* mutant strains compared to the reference strains within the experimental period, presumably as a consequence of a decreased carbon flux through the lower part of glycolysis and the TCA cycle in these mutants (Siedler et al. 2012).

*C. glutamicum* possesses two glyceraldehyde 3-phosphate dehydrogenases (GAPDH), but only GapA functions in the glycolytic direction (Omumasaba et al. 2004). Thus, a deletion of the corresponding gene theoretically should result in a cyclization of the PPP. The fact that the MHB per glucose yield of the strain ∆*gapA*/pEKEx2-*Lbadh* (7.9 mol/mol) was higher compared to the strain ∆*pfkA*/pEKEx2-*Lbadh* and corresponded to 66 % of the maximal value of 12 mol NADPH per mole of glucose indicated a more extended cyclic operation of the PPP in the ∆*gapA* mutant compared to the ∆*pfkA* mutant. The maximal value for a complete oxidation of glucose in the PPP was not reached because 25 % of the glucose carbon was lost by reduction of glyceraldehyde 3-phosphate to glycerol. Taking this loss into account, only 9 mol_MHB_ mol_glucose_^−1^ could be achieved maximally. The experimental yield of 7.9 mol_MHB_ mol_glucose_^−1^ corresponds to 88 % of this value and is 46 % above the best yields reported so far (Chin and Cirino 2011; Siedler et al. 2011, 2012). Future yield optimization could be achieved by deletion of the gene encoding glycerol 3-phosphatase. Such a deletion was recently shown to prevent glycerol formation, which predominantly occurs in fructose-utilizing *C. glutamicum* strains (Lindner et al. 2012).

The strongly reduced biotransformation rate of the strain Δ*gapA*/pEKEx2-*Lbadh* was probably a consequence of the diminished capability for glucose uptake. In a Δ*gapA* mutant, no PEP should be formed during glucose catabolism and consequently, glucose uptake via the PTS should be impossible. PTS-independent glucose uptake has recently been described for *C. glutamicum.* It involves the inositol transporters IolT1 and IolT2 which also function as low-affinity glucose permeases (Lindner et al. 2011). Subsequent phosphorylation of glucose to glucose 6-phosphate is catalyzed either by an ATP-dependent glucokinase encoded by *glk* (Park et al. 2000) or by the polyphosphate- or ATP-dependent glucose kinase PpgK (Lindner et al. 2010). It can be assumed that glucose uptake during biotransformation with the Δ*gapA* mutant occurs via this alternative pathway, as the observed glucose consumption rate of 2.5 nmol min^−1^ mg_cdw_^−1^ (Table 5) at glucose concentrations >10-fold above the apparent *K*_s_ values of IolT1 and IolT2 (2.8 and 1.9 mM, respectively) is in the range determined for PTS-independent glucose uptake at 1 mM glucose (0.7 nmol min^−1^ mg_cdw_^−1^) (Lindner et al. 2011). Overexpression of either *iolT1* or *iolT2* together with *ppgK* was shown to allow almost wild-type growth rates in a PTS-negative mutant (Lindner et al. 2011) and thus would probably also allow higher biotransformation rates of a Δ*gapA* mutant. Alternatively, expression of the glucose facilitator gene *glf* from *Zymomonas mobilis* could help to increase glucose uptake (Weisser et al. 1995; Parker et al. 1995).

Overall, we could demonstrate the potential of *C. glutamicum* for NADPH-dependent reductive whole-cell biotransformation and show that deletion of either *pfkA* or *gapA* is beneficial to improve the NADPH per glucose yield, presumably by cyclization of the PPP.
